# Differential Attraction of Summer and Winter Morphs of Spotted Wing Drosophila, *Drosophila suzukii*, to Yeasts

**DOI:** 10.1007/s10886-025-01561-x

**Published:** 2025-02-05

**Authors:** Rory Jones, Matthew R. Goddard, Paul E. Eady, David R. Hall, Daniel P. Bray, Dudley I. Farman, Michelle T. Fountain

**Affiliations:** 1 School of Life Sciences, University of Lincoln, Lincoln, LN6 7DL UK; 2 NIAB, New Road, East Malling, Kent, ME19 6BJ UK; 3 Natural Resources Institute, University of Greenwich, Chatham Maritime, Kent, ME4 4TB UK

**Keywords:** Invasive pest, IPM, Morphotypes, Olfactory attraction, SWD, Microbes

## Abstract

*Drosophila suzukii* is a pest of soft and stone fruits that is attracted to yeast volatile metabolites. *Drosophila suzukii* has distinct summer and winter morphs which are found in different habitats. Complex communities of yeasts likely differ between habitats and thus we hypothesized morphs differ in their attraction to yeast volatiles from different species and combinations of species. We presented *D. suzukii* with yeast species in isolation and in combinations, as either post-culture mixes or co-cultured, and measured activity of the flies in laboratory choice tests with a Locomotor Activity Monitor as a proxy for attraction. *Candida zemplinina* was more attractive to winter than summer morphs when cultured in both sterile strawberry juice (SSJ) and artificial culture media (YPD). No significant correlations were found between principle component scores derived from single yeast volatile profiles and fly activity. There was also differential attraction of morphs to certain yeast mixes, most notably post SSJ culture mixes of *Hanseniaspora uvarum* and *C. zemplinina* were consistently more attractive to winter morphs. Mixtures of *Metschnikowia pulcherrima* + *Pichia pijperi* + *H. uvarum* in SSJ and *M. pulcherrima* + *P. pijperi* yeasts in YPD were also preferred by winter morphs, whilst summer morphs were attracted to SSJ co-cultures of *M. pulcherrima* + *H. uvarum*. However, co-culturing yeasts did not enhance attraction compared to post-culture mixes of constituent yeasts for either morph. Differential attraction of morphs to certain yeasts suggests options for new bait formulations in integrated pest management strategies may be tailored to better target morphs.

**Supplementary Information:**

The online version contains supplementary material available at 10.1007/s10886-025-01561-x.

## Introduction

Spotted wing drosophila, *Drosophila suzukii* (Matsumura) (Diptera: Drosophilidae), oviposits in ripening fruits and the resultant larvae feed on the fruits causing fruit degradation and yield loss, and as such is a major pest for the soft-fruit industry (De Ros et al. 2020). *Drosophila suzukii* is an invasive pest species that has spread from its native range in South East Asia and now affects fruit production in most northern temperate regions (Asplen et al. 2015; Rota-Stabelli et al. 2013), including the UK (Harris and Shaw 2014).

*Drosophila suzukii* is polyphenic; it has both winter and summer morphs which can occupy different habitats. Summer morphs occupy crops and semi-natural habitats, whereas winter morphs generally leave cropping areas seeking shelter in other habitats like woodlands and hedgerows towards the end of the fruit growing season (Buck et al. 2023; Stockton et al. 2019; Zerulla et al. 2015). Temperature is the main cue for the transition between morphs (Leach et al. 2019), and the key phenotypic differences are that winter morphs are larger and express a greater extent of melanization (Wallingford and Loeb 2016). As temperatures increase during spring, winter morphs become more active and transition to areas with fruit crops and females develop ovaries and eggs (Panel et al. 2018).

Clymans et al. (2019) reported *D. suzukii* female winter morphs were more attracted to apple cider vinegar than summer morphs. Geosmin is a volatile that is produced by some fungi including those that cause post-harvest fruit rots, such as *Penicillium* which has been identified in the gut of winter-morph *D. suzukii* (Boerjesson et al. 1993; Fountain et al. 2018; Mattheis and Roberts 1992; Sunesson et al. 1995). Geosmin repels summer but not winter *D. suzukii* morphs (Conroy et al. 2024; Kirkpatrick et al. 2018). *Drosophila suzukii* morphs also differ in their response to other chemical stimuli at certain concentrations, with methyl 2-aminobenzoate (methyl anthranilate) and butyl 2-aminobenzoate (butyl anthranilate) being repellent to winter but not summer morphs, and vice versa for butyl acetate and methyl 2-hydroxybenzoate (methyl salicylate) (Conroy et al. 2024). Further, differences in olfactory preference between summer and winter morphs may be driven in part by the differing resource requirements of mated and un-mated females; for example, Karageorgi et al. (2017) found *D. suzukii* were attracted to over-ripe fruit for feeding but ripening fruit for oviposition. These observations suggest that the two morphs perceive and respond to environmental cues differently.

Yeasts are an important source of nutrients for *Drosophila* as they provide a source of protein critical for egg development (Plantamp et al. 2016). Female flies prefer to oviposit on yeast-colonized substrates (Oakeshott et al. 1989) and larvae develop quicker on a diet containing yeast (Bellutti et al. 2018; Meshrif et al. 2016). Diets without microbes cause early death in *D. suzukii* which is probably due to protein starvation (Bing et al. 2018; Hardin et al. 2015). It is therefore likely that selection has operated to increase *D. suzukii’s* ability to locate yeasts via stimuli such as metabolic volatiles released from yeasts (Günther et al. 2019; Jones et al. 2021). In line with this, the majority of effective bait formulations for *D. suzukii* are based on yeasts and their metabolic products such as mixes of red wine and vinegar, apple cider vinegar alone, sugar and *Saccharomyces cerevisiae* mixtures (Iglesias et al. 2014; Landolt et al. 2012), and synthetic baits containing ethanol, acetic acid, methionol, and acetoin (Cha et al. 2013, 2015, 2017). Yeasts can also be used as phagostimulant baits in combination with insecticides which stimulate *D. suzukii* to feed and thus ingest toxic baits (Mori et al. 2017). For example, the addition of *Hanseniaspora uvarum* to insecticide products results in comparable levels of control of *D. suzukii* to those conventional insecticides alone, but with up to a 90–96% reduction in the amount of insecticide used (Bjeljac et al. 2024; Noble et al. 2021).

Different yeast species produce distinct metabolic volatile profiles as they grow which also differ depending on the growth substrate (Bueno et al. 2020; Günther et al. 2015; Scheidler et al. 2015). Experimental work shows *D. suzukii* summer morphs are attracted to *H. uvarum*, *Hanseniaspora opuntiae*,* Metschnikowia pulcherrima*, *Pichia pijperi*, *Pichia terricola*,* Candida zemplinina*, *Candida californica*,* Saccharomycopsis vini*, and *S. cerevisiae* (Biasazin et al. 2022; Bueno et al. 2020; Castellan et al. 2024; Erdei et al. 2022; Jones et al. 2021; Kleman et al. 2022; Lasa et al. 2019; Noble et al. 2019; Rehermann et al. 2022; Scheidler et al. 2015). Some species of yeast appear to be more attractive than others. For example, *H. uvarum* and *M. pulcherima* are more attractive to *D. suzukii* than *S. cerevisiae* (Chakraborty et al. 2022; Jones et al. 2021; Scheidler et al. 2015). However, current *Drosophila* yeast-based baits often only use *S. cerevisiae.*

Natural communities of yeasts on fruit, and likely in other substrates in surrounding non-managed habitats, comprise tens to hundreds of species (Fountain et al. 2018; Jones et al. 2022b; Taylor et al. 2014) and, importantly, the types of species in communities differ between habitats (Morrison-Whittle and Goddard 2018). Thus, both morphs of *D. suzukii* are naturally exposed to different mixes of volatiles from varying yeast communities in separate habitats. Experimental work has shown that combinations of volatiles from *H. uvarum*,* M. pulcherrima*,* P. pijperi*, and *C. zemplinina*, mixed after each has been cultured separately, are attractive to *D. suzukii* summer morphs, although not significantly more so than volatiles from *H. uvarum* alone (Jones et al. 2021). Also, *H. uvarum* combined with *P. terricola* was no more attractive than each constituent yeast alone (Erdei et al. 2022). Analyses of volatile thiol production by *S. cerevisiae* and *Pichia kluyveri* separately and when co-cultured, i.e. grown together in mixed communities, provided evidence that co-cultured yeast species may interact to affect the overall types and amounts of metabolic volatiles produced compared to those produced by each member of the community separately (Anfang et al. 2009). Thus, co-cultured yeasts may better emulate the natural situation and produce volatile profiles to which *D. suzukii* is more attuned. For example, *D. melanogaster* is more attracted to certain yeast-bacteria co-cultures compared to the same microbes cultured separately and then combined (Fischer et al. 2017).

Studies that have evaluated the attraction of *D. suzukii* winter morphs to yeasts are limited in number. Attraction to *M. pulcherrima*, *S. cerevisiae*, *H. uvarum*, *P. terricola* and combinations of the latter two were consistent between winter and summer morphs (Erdei et al. 2022; Kirkpatrick et al. 2018; Wong et al. 2018). However, we are not aware of any studies that have assessed the specific attraction of *D. suzukii* winter morphs to other yeasts and mixes of yeasts. The development of novel control measures that more effectively attract and trap winter morphs could potentially reduce pest populations at the start of the growing season and provide powerful pest control measures.

To address this gap in knowledge we tested three hypotheses using laboratory activity monitors to quantify fly behavior: (1) that *D. suzukii* female summer and winter morphs differ in their attraction to volatiles from single yeast types cultured in isolation; (2) that *D. suzukii* female summer and winter morphs differ in their attraction to volatiles from mixes of different yeast types; and (3) that some co-cultured yeasts will be more attractive than mixes combined post-culture or single yeast alone for both *D. suzukii* morphs.

## Methods and Materials

### Cultures of *Drosophila suzukii*

The strain of *D. suzukii* used derived from flies collected near Trento, Italy and maintained in culture, which had not been refreshed with wild caught flies, for seven years prior to use. Summer-morph flies were housed in BugDorm cages (32.5 × 32.5 × 32.5 cm; MegaView Science Co., Ltd., Taichung 407008, Taiwan) at 89% relative humidity provided by damp blue absorbent paper on the roof and base of the cages at 22 ± 1.5 °C with a 16:8 h L: D photoperiod (Shaw et al. 2018). *Drosophila* Quick Mix Medium blue (Blades Biological Ltd., Edenbridge, Kent, UK) sprinkled with dried baker’s yeast (*S. cerevisiae*) was used to rear summer-morph flies (Jones et al. 2022a). Cages were provisioned with thawed frozen raspberries weekly. Winter morphs were generated by transferring summer-morph adult flies from culture cages to square or circle-based *Drosophila* Bottles (177 mL, Fisherbrand, Fisher Scientific, Loughborough, UK) filled with 50 mL cornmeal media (1% agar, 9% sugar, 9% pre-cooked ground maize, 2% baker’s yeast, 5% malt, 1% soy flour, 0.3% propionic acid and 0.3% methyl 4-hydroxybenzoate pre-dissolved in 70% ethanol). Flies were left to oviposit and larvae to develop for seven days whereupon adult flies were removed, and the bottles maintained at 10 °C and 0:24 h L: D photoperiod. Before use in experiments, adult winter-morph *D. suzukii* were transferred to *Drosophila* bottles containing 50 mL of *Drosophila* Quick Mix Medium sprinkled with yeast and were then acclimatized to 22 °C and 16:8 h L: D photoperiod over a three-day period.

### Yeast Cultures

Yeast species were from the Goddard culture collection at the University of Lincoln, all originating from grapes or the fermentation of grapes (Supplementary Material, Table S1). These yeast strains were selected based on their previous use in attraction tests to *D. suzukii* (Jones et al. 2021). Yeast were grown at 30 °C with 180 rpm shaking and were pre-cultured for 24 h in YPD media (1% yeast extract, 2% peptone and 2% dextrose, sterilized by autoclaving) whereupon optical density (600 nm) was assessed and 1 × 10^6^ yeast cells per mL were transferred to either 20 mL of YPD media or sterile strawberry juice (var. Driscoll’s^®^ Katrina) sterilized by 0.2 μm filtration (SSJ), and cultured for 48 h at 30 °C (*N* = 4 per yeast treatment). The media was centrifuged at 4,500 rpm for 10 min to collect cells, and the supernatant containing yeast metabolites was decanted and stored frozen prior to use in fly choice tests (Jones et al. 2021). Yeasts were cultured either alone or co-cultured with other species. Where yeasts were co-cultured, cultures were inoculated with an equivalent number of cells (1 × 10^6^ cells per mL) from each yeast species.

### Laboratory Multiple-Choice Tests

To assess the attraction of female *D. suzukii* summer and winter morphs to metabolites in yeast post-culture media, choice tests were established in a BugDorm cage (475 × 475 × 475 mm) using a 32-channel modified LAM10H Locomotor Activity Monitor (TriKinetics Inc., Waltham, MA) with open ended 25 mm diameter tubes arranged in an 8 × 4 matrix (Supplementary Material Fig. S1; Noble et al. 2019). Each tube in the activity monitor had a planar array of three infra-red beams 23 mm from the base of the tubes and the apparatus was set up at an angle of 20° from horizontal to prevent media running past the infra-red beams and registering false counts. Each tube was baited with 0.2 mL of the corresponding yeast post-culture media or control at the base of the tubes. Conditions inside the cage were 22 °C and 89% relative humidity with a 16:8 h L: D photoperiod. Flies were anesthetized using CO_2_ for up to a maximum of 6 min, sex determined, and then starved for approximately 1–2 h prior to being added to the cage. Until flies were sexed, females had access to males and as such were considered mated as insemination can occur within 24 h of emergence (Revadi et al. 2015). Groups of 3–10-day old female *D. suzukii* (200 summer morphs or 130–140 winter morphs) were added to the cage and acclimatized before the start of the experiment for 5 min for summer and 2 h for winter morphs, reflecting the differential times taken for the morphs to become active (start to register counts on the Activity Monitor). Flies were free to enter/exit tubes at will, when they crossed the inferred beams (located 23 mm from the base of the tubes) in either direction each pass over a beam registered as a count in the corresponding tube. Activity counts were used as a proxy for attraction compared to the other choice options available to the flies (Noble et al. 2019). Using activity counts assumes that the increased levels of *D. suzukii* activity in tubes is because the treatments are more attractive and hence activity is used as a proxy for attraction. Experiments lasted 24 h and the total numbers of counts in six four-hour periods for the duration of the experiment were recorded.

Three multiple-choice experiments were carried out for each morph separately: (1) attractiveness of post-culture media from yeasts cultured in isolation (*H. uvarum*, *M. pulcherrima*, *P. pijperi*, *C. zemplininia* and *S. cerevisiae*); (2) attractiveness of blends of post-culture media from yeasts cultured in isolation (*H. uvarum + C. zemplininia*, *M. pulcherrima + H. uvarum*, *M. pulcherrima + P. pijperi* and *M. pulcherrima + P. pijperi + H. uvarum*); and (3) attractiveness of media from co-cultured mixes of different yeast types (*H. uvarum* + *C. zemplininia* and *M. pulcherrima* + *H. uvarum*) compared with the same combinations blended post-culture (Supplementary Material, Table S2). All yeasts treatments were cultured with YPD and SSJ media. Distilled water, a commercially-available bait Combi-protec (5% v/v solution; Andermatt UK, Hove, UK), and growth media with no yeasts were included as controls in all experiments. Additionally, for yeast combination experiments, *H. uvarum* alone was also included (Supplementary Material Table S2). For each experiment, all treatments were present in randomized order in each of the four rows of the Locomotor Activity Monitor (Supplementary Material Fig. S1). Three runs, each containing four biological replicates of supernatant from yeast cultures, were carried out for each experiment resulting in *N* = 12 replicates for each treatment. Summer and winter morphs were tested for attraction in separate experiments, and the activity data were subsequently converted to proportions to allow comparison between morphs due to the differing total numbers of flies used for summer and winter-morph experiments. Where comparisons between treatments within each morph were made, analysis was done on count data and not proportions.

### Analysis of Volatiles

Headspace volatiles from the yeast post-culture supernatants (5 mL and *N* = 3 per sample) were collected by solid-phase microextraction (SPME) using SPME needles coated with divinylbenzene/Carboxen/PDMS (grey; 100 µ; Supelco, Gillingham, Dorset, UK) over a 30-min exposure period. The fibers were desorbed in the injector of a gas chromatograph (Varian CP3700; Agilent, Manchester, UK) coupled to a mass spectrometer (Saturn 2200; Agilent) operated in EI mode (GC-MS). The GC had a fused silica capillary column (30 m x 0.25 mm i.d. x 0.25 μm film thickness) coated with polar DBWax (Agilent) with helium as carrier gas (1.5 mL/min) and splitless injection (220 °C). The oven temperature was held at 40 °C for 2 min and then programmed at 10 °C/min to 250 °C and held for 5 min. Compounds with peak areas ≥ 0.1% of the total were identified according to their mass spectra and confirmed by comparison of their retention indices relative to the retention times of n-alkanes and mass spectra with those of authentic standards.

### Statistical Analyses

All statistical analyses were carried out in R version 3.6.1 (R Core Team 2019) with the MASS (Venables and Ripley 2002) and emmeans (Lenth et al. 2019) packages. To determine if the preference of summer- and winter-morph females to yeasts differed, the choice data from the relevant separate summer- and winter-morph experiments were combined and analyzed using binomial logistic regressions. Data for the six four-hour periods in each experiment are shown in the Supplementary Material. To control for differences in the number of winter- and summer-morph flies used in the experiments, the number of beam-breaks per replicate (i.e. number of flies responding to a stimuli) were effectively expressed as a proportion of all other beam-breaks (i.e. number of flies not responding to a specific treatment) by binding these two vectors into a single object. In this way, the binomial logistic regression is a weighted regression. As such this analysis looked at proportional data and therefore relative attraction. In the logistic regressions, treatment (yeast cultures or controls; Table S2), experiment batch (replicate run) and fly morphological type were treated as fixed factors with time (six four-hour periods) a covariate. The quasibinomial family was chosen for the logistic regression as the data were over-dispersed (dispersion calculated as residual deviance divided by residual degrees of freedom = 14.15–22.67). Significance was assessed using a *F*-test of the change in residual deviance following model simplification. Following Crawley (2013), the significance (or otherwise) of the factors, covariates, and interactions were assessed and either retained or deleted from the model (depending on their significance) in a step-wise manner, starting with the highest-order interaction effects to produce the minimal adequate model.

To investigate the effect of co-culturing yeasts on attraction, the data from the experiments with co-cultured yeasts were also analyzed separately for each morph. Count data (total number of counts across six four-hour periods) were analyzed using generalized linear models (GLMs) with treatment and experimental batch (replicate run) as fixed factors with time as a covariate. The negative binomial family was chosen for the GLM’s as the data were over-dispersed (dispersion = 13.46–27.19). Significance was assessed using ANOVA following model simplification as per Crawley (2013).

The volatile profiles obtained from the supernatant from single yeast cultures were analyzed using principal component analysis (PCA) on TIC peak areas with a rotation applied to the principal components (Wehrens 2011), using ‘prcomp’. PC1 and PC2 were investigated against peak areas of specific volatile chemicals obtained from GC-MS analysis to the most influential constituent volatiles for separating yeast volatile profiles. PC1 and PC2 were also correlated to attractiveness, both total counts after 4 and 24 h, to summer and winter morphs using Pearson correlation coefficients (Crawley 2013).

## Results

### Attraction of *Drosophila suzukii* Summer and Winter Morphs to Volatiles from Yeast Types Cultured in Isolation

**Fig. 1:**
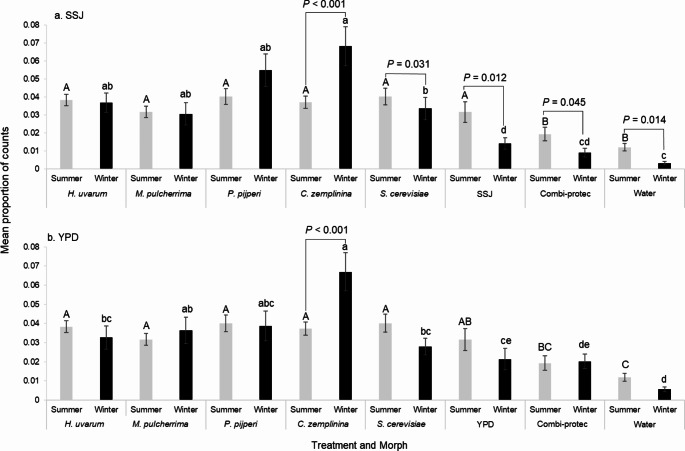
Mean proportion of activity counts (± SE; *N* = 12) over 24 h, as a proxy for attraction of summer (grey bars) and winter (black) morphs of *Drosophila suzukii* for single yeasts (*Hanseniaspora uvarum*, *Metschnikowia pulcherrima*, *Pichia pijperi*, *Candida zemplininia* and *Saccharomyces cerevisiae* grown in (**a**) sterile strawberry juice (SSJ) or (**b**) yeast peptone dextrose (YPD) media, alongside growth media control (either SSJ or YPD), Combi-protec, and distilled water control. Summer and winter morphs were tested for attraction in separate experiments. Proportions calculated as number of counts for each treatment divided by total number of counts. *P*-values above black bars show significance different of attraction between morphological type. Upper case (summer) and lower-case letters (winter) show significant differences in attraction between treatments for the same morph type determined by Tukey tests post hoc (*P* < 0.05). For full graphical representations of the data see Supplementary Material Figs. S2; S3)

For comparison between winter and summer morphs, data (beam-breaks (or counts) per replicate expressed as a proportion of all other beam-breaks) from the separate experiments for each morph were analyzed by binding the two values together into a single weighted object (Crawley 2013) (Fig. 1a and b; Supplementary Material Figs. S2 and S3). *Drosophila suzukii* morphs differed in their attraction to yeasts cultured in both SSJ and YPD as revealed by a significant interaction between treatment and morph (Δ deviance = 1227.5, *df* = 7, *P* < 0.001 and Δ deviance = 485.15, *df* = 7, *P* = 0.004, respectively; Fig. 1a and b). There were no significant effects of time or experimental batch on attraction. *Candida zemplininia* was more attractive to the winter than summer morphs when cultured both in both SSJ and YPD (*P* < 0.001) and was the most attractive treatment to winter morphs overall. There was greater attraction of summer morphs to *S. cerevisiae* when cultured in SSJ (*P* = 0.031) but not YPD media (*P* = 0.12). For the SSJ media experiment, Combi-protec, as well as the water and SSJ controls were more attractive to the summer morphs (*P* = 0.012, *P* = 0.045 and *P* = 0.014, respectively; Fig. 1a). These data and analyses are in line with the first hypothesis that *D. suzukii* female summer and winter morphs differ in their attraction to volatiles for some (*C. zemplininia* and *S. cerevisiae*) single yeast types cultured in isolation, with *C. zemplininia* showing the most marked attraction to winter morphs, which was preferred over *S. cerevisiae* when cultured in either SSJ (*P* = 0.049), albeit very marginally, or YPD (*P* = 0.022) and *H. uvarum* in YPD (*P* = 0.043). Summer morphs did not prefer particular yeast species (Fig. 1; S3).

### Analyses of Volatiles from Single Yeasts

**Fig. 2:**
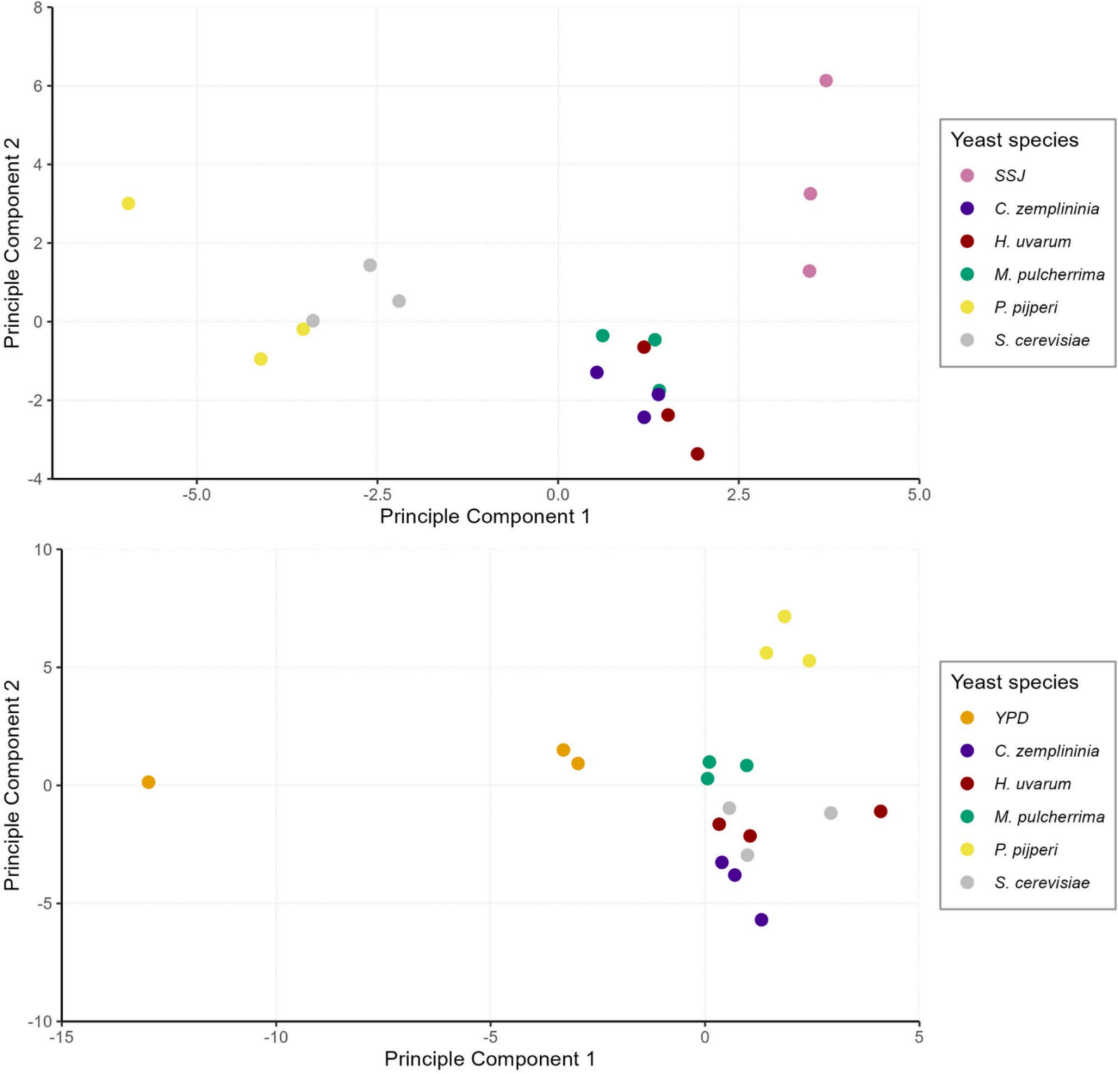
Principal component analysis (PCA) of the volatile profile from single yeasts (*Hanseniaspora uvarum*, *Metschnikowia pulcherrima*, *Pichia pijperi*, *Candida zemplininia* and *Saccharomyces cerevisiae; N* = 3) cultured in sterile strawberry juice (SSJ) (top) and yeast peptone dextrose (YPD) media (bottom)

The relative amounts of compounds from single yeast types cultured in isolation collected by SPME and analyzed by GC-MS are shown in Supplementary Material Tables S3 and S4. PCA of the volatile profiles show separation for the different yeast types across both growth media (Fig. 2). For volatiles from single yeast species grown in SSJ and YPD the first two principal components explained 51.23% and 38.3% of the variance in the volatile data respectively.

For SSJ media, volatiles from the control were separated from the yeast treatments in PCA plots, and the yeast treatments differentiated into two clear groups with *P. pijperi* and *S. cerevisiae* differentiated from *H. uvarum*, *M. pulcherima* and *C. zemplininia* (Fig. 2). PC1 was positively correlated with peak areas obtained from GC-MS analyses for benzaldehyde (Pearson’s correlations, *P* < 0.05) although this was also present in the SSJ alone. PC2 separated SSJ from *H. uvarum*, *M. pulcherima* and *C. zemplininia* and was positively correlated with methyl hexanoate, (*E*)-2-hexenyl acetate and hexanoic acid (*P* < 0.05). For experiments with YPD media, yeast treatments and the control were again separated on PCA plots and volatiles from *P. pijperi*, and less so for *M. pulcherima*, formed distinct groups (Fig. 2). Separations on PC1 were positively correlated with peak areas obtained from GC-MS analysis for ethanol, 2/3-methylbutanol and 2-phenylethanol (*P* < 0.05), PC2 was positively correlated with peak areas for ethyl acetate, ethyl butanoate and 2/3-methylbutyl acetate (*P* < 0.05).

Despite differential attractiveness of *D. suzukii* winter morphs to some single yeast species cultured in isolation reported by the activity monitor and PCA plots showing separation of the volatile profiles of these culture products, there were no significant correlations between PC1 and PC2 scores and fly activity (total counts after 4 h and 24 h), for winter morphs when the yeasts were cultured in SSJ or YPD (Table S5).

### Attraction of *Drosophila suzukii* Summer and Winter Morphs to Volatiles from Mixtures of Yeast Species

#### Attraction to Post-Culture Yeast Mixes

**Fig. 3:**
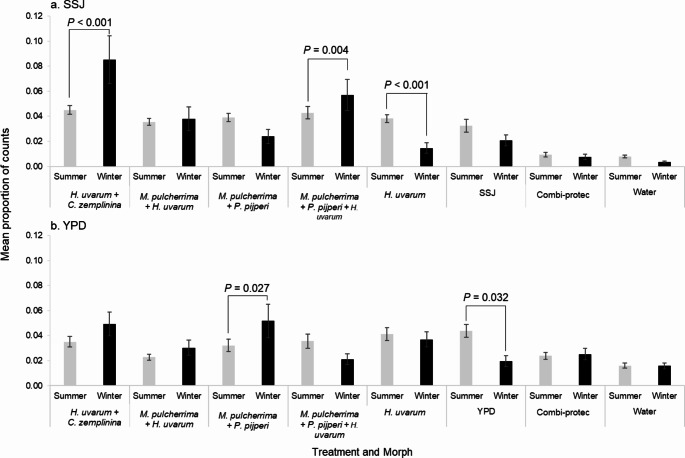
Mean proportion of activity counts (± SE; *N* = 12) recorded over 24 h as a proxy for attraction of *Drosophila suzukii* summer (grey bars) and winter (black) morphs to post-growth mixes of different yeast species (*Hanseniaspora uvarum* + *Candida zemplininia*,* Metschnikowia pulcherrima* + *H. uvarum*,* M. pulcherrima* + *Pichia pijperi* and *M. pulcherrima* + *P. pijperi* + *H. uvarum*) grown in (**a**) sterile strawberry juice (SSJ) and (**b**) yeast peptone dextrose (YPD) media. Single yeast *H. uvarum*, sterile growth media, Combi-protec, and distilled water controls are included. *P*-values above black bars show significant differences in attraction between morphological type. For full graphical representations of the data see Supplementary Material Figs. S4; S5)

The winter and summer morphs of *D. suzukii* differed in their attraction to post-culture mixes of yeasts in both SSJ and YPD media, as revealed by a significant interaction between treatment and morph (Δ deviance = 1062.7, *df* = 7, *P* < 0.001 and Δ deviance = 366.82, *df* = 7, *P* = 0.010, respectively; Fig. 3, Supplementary Material Figs. S4 and S5). There was no significant effect of time (six four-hour periods) or experimental batch on *D. suzukii* activity in these experiments. Post-hoc tests for differences in attractiveness to the two morphs in SSJ showed post-culture mixes of *H. uvarum* + *C. zemplinina* and *M. pulcherrima* + *P. pijperi* + *H. uvarum* were more attractive to winter than summer-morph females (*P* < 0.001 and *P* = 0.004, respectively; Fig. 3a). The *H. uvarum* control also showed differential attraction of morphs, being more attractive to the summer-morph (*P* < 0.001). In YPD, only the *M. pulcherrima* + *P. pijperi* mix showed differential attraction between morphs and was more attractive to winter than to summer morphs (*P* = 0.027). The YPD control was more attractive to summer morphs (*P* = 0.032) (Fig. 3b). The data and analyses are thus in line with the hypothesis that *D. suzukii* female summer and winter morphs differ in their attraction to post-culture blended volatiles from some but not all mixes of different yeast types (Fig. 3). Overall, the post-culture mix of *H. uvarum* + *C. zemplinina* grown in SSJ was the most attractive to winter morphs of *D. suzukii.*

#### Attraction to Co-Cultured Yeast Mixes

**Fig. 4:**
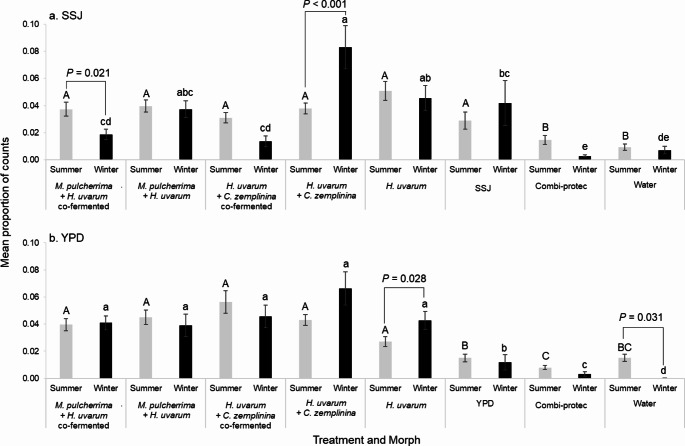
Mean proportion of activity counts (± SE; *N* = 12) used as a proxy for attraction of female *Drosophila suzukii* summer (grey bars) and winter (black) morphs to combinations of co-cultured or post-cultured mixes of different yeasts (*Metschnikowia pulcherrima* + *Hanseniaspora uvarum* and *H. uvarum* + *Candida zemplininia*) grown in (**a**) sterile strawberry juice (SSJ) and (**b**) yeast peptone dextrose (YPD) media. Single yeast *H. uvarum*, growth media, Combi-protec, and distilled water controls are included. Summer and winter morphs were tested for attraction in separate experiments. Proportions were calculated as number of counts for each treatment divided by total number of counts. *P*-values above black bars show significance differences in attraction between morphological types. Upper case (summer) and lower-case letters (winter) show significant differences in attraction between treatments for the same morph type determined by Tukey tests post hoc (*P* < 0.05). For full graphical representations of the data see Supplementary Material Figs. S6; S7)

*Drosophila suzukii* morphs differed in their attraction to yeasts co-cultured in both SSJ and YPD, as revealed by a significant interaction between treatment and morph (Δ deviance = 780.73, *df* = 7, *P* < 0.001 and Δ deviance = 691.14, *df* = 7, *P* < 0.001, respectively, Fig. 4, Supplementary Material Figs. S6, S7). Again, there was no significant effect of time (six four-hour periods) or experimental batch on attraction of *D. suzukii* (*P* = 1). There was only one instance where co-cultured yeasts differed in attraction between morphs: co-cultured *M. pulcherrima* + *H. uvarum* in SSJ were more attractive to summer than winter morphs (*P* = 0.021), but there was no difference in attraction between morphs for SSJ post-culture blends of volatiles from *M. pulcherrima* + *H. uvarum* (*P* = 0.25). As in the previous trial, SSJ post-culture blends of *H. uvarum* + *C. zemplinina* were more attractive to winter than summer morphs (*P* < 0.001), and the most attractive to winter morphs overall. However, there were some inconsistencies in preferences for yeast between the morphs in different experiments: for example, *H. uvarum* cultured in YPD and SSJ showed differential attraction to morphs in some but not all experiments (Figs. 1, 3 and 4).

### Comparison of Attraction to Co-Cultured and Post-Culture Yeast Mixes

Data from the experiments with co-cultured yeasts (Supplementary Material Figs. S6, S7) were also analyzed separately for each morph, to test the hypothesis that co-cultured volatile profiles are more attractive to both *D. suzukii* morphs than profiles from single yeasts combined post-culture or from single yeasts. Across both winter and summer morphs experiments for both culture media there was a significant effect of treatment on activity counts (Δ deviance = 95.42–138.13; *df* = 7; *P* < 0.001). Experimental batch was also significant across all experiments (Δ deviance = 12.84–72.73; *df* = 2; *P* < 0.003). Time influenced *D. suzukii* activity significantly in winter-morph SSJ experiments only (Δ deviance = 21.40; *df* = 7; *P* < 0.001; *P* > 0.11 for the rest), and there was no significant interaction between time and treatment for any experiment (*P* > 0.11).

For females of both morphs there was no greater preference for co-cultured yeasts, *M. pulcherrima* + *H. uvarum* or *H. uvarum* + *C. zemplininia*, in either medium. In fact, for winter morphs, *H. uvarum* + *C. zemplininia* co-cultured in SSJ was less attractive than the post-culture mixture of the two (Tukey *P* < 0.001; Fig. 4a), and co-cultured *M. pulcherrima + H. uvarum* was less attractive than the post-culture mixture, but not significantly so (Fig. 4a). Furthermore, neither of the co-cultured yeasts was more attractive to winter-morph *D. suzukii* than *H. uvarum* alone in either medium, with both significantly less attractive than *H. uvarum* alone in SSJ (*P* = 0.001; Fig. 4). For summer morphs, there was no significant difference in attraction between any yeast treatments grown in SSJ and the SSJ media control (Fig. 4a).

However, there were significant differences in attraction across the yeast treatments grown in SSJ for the winter-morph (Fig. 4a): post-culture mixes of volatiles from *H. uvarum* + *C. zemplinina* were most preferred by winter morphs and were significantly more attractive than all other treatments apart from the single *H. uvarum* and *M. pulcherrima + H. uvarum* post-culture mix treatments. There was significant variance in attraction among the rest of the yeast treatments (Fig. 4a). When grown in YPD, there was no difference in attractiveness between any yeast treatments for both morphs (Fig. 4b). However, most yeast treatments cultured in both SSJ and YPD were more attractive than the water or Combi-protec controls (*P* < 0.0014), with the only exception being co-cultures of *H. uvarum* with either *M. pulcherrima*, or *C. zemplinina* for winter morphs in SSJ (Fig. 4a). There is no evidence that co-cultures of different yeasts are more attractive than post-culture mixes of yeasts cultured individually.

## Discussion

Yeasts are attractive to *D. suzukii* and have the potential to be used as baits (Kleman et al. 2022; Noble et al. 2021; Rehermann et al. 2022; Scheidler et al. 2015; Spitaler et al. 2022). Despite some evidence that olfactory attraction varies between *D. suzukii* morphs (Clymans et al. 2019; Kirkpatrick et al. 2018) there is limited data investigating whether attraction to yeasts differs. The development of novel control measures that can more effectively target winter morphs could reduce pest populations at the start of the growing season and provide potentially powerful pest control measures.

The data generated here support the first two hypotheses: (1) *D. suzukii* female summer and winter morphs differ in their attraction to volatiles from cultures of single yeast types, with *C. zemplininia* showing the most marked attraction to winter morphs, but we could not identify any volatiles that correlated with this; and (2) *D. suzukii* female summer and winter morphs differ in their attraction to volatiles from mixes of different yeast types, with the *H. uvarum and C. zemplinina* post-culture mix in SSJ being the most attractive to winter morphs. The third hypothesis, that co-cultured yeasts will be more attractive than mixes combined post-culture or single yeasts alone was not supported for either morph of *D. suzukii*.

Even though single yeast species cultures produced different volatile organic compound profiles, our analyses did not show any compounds that correlate with attractiveness to winter-morph *D. suzukii*. The context of volatiles is important for *D. simulans* attraction to yeasts (Günther et al. 2015), and it may be that other less abundant volatiles play an important role in modulating attraction, or that other cues like taste and/or color are also needed to attract *D. suzukii*. Volatile chemicals that differed between yeasts from our samples included benzaldehyde, ethanol, 2/3-methylbutanol, 2-phenylethyl acetate, ethyl butanoate and 2/3-methylbutyl acetate, which are all common yeast fermentation products (Bueno et al. 2020; Günther et al. 2015; Scheidler et al. 2015). Bueno et al. (2020) found greater levels of 3-methylbutyl acetate (isoamyl acetate) from samples containing *Pichia* species, which was less attractive than samples of *H. uvarum* to *D. suzukii.* We also found increased levels of 2/3-methylbutyl acetate in samples of *P. pijperi* compared to *H. uvarum* (Supplementary Material, Tables S3 and S4). Previous work showed that when combined with acetic acid and ethanol, 3-methylbutyl acetate reduced attractiveness to *D. suzukii* (Cha et al. 2012).

Despite there being no difference in preference for most of the single or combinations of yeast species between the morphs, in certain cases a preference was observed. This suggests that in certain circumstances attraction to yeast species may vary. Overall, winter morphs were more attracted to *C. zemplinina* post-culture media either alone or when mixed with *H. uvarum* post-culture media than summer morphs. Additionally, co-cultured *M. pulcherrima* + *H. uvarum* in SSJ were marginally more attractive to *D. suzukii* summer than winter morphs (Fig. 4). Post-SSJ culture mixtures of *M. pulcherrima* + *P. pijperi* + *H. uvarum*, and *M. pulcherrima* + *P. pijperi* in YPD, were more attractive to female winter than summer morphs but to lesser extents. In contrast, *S. cerevisiae* and co-cultured *M. pulcherrima* + *H. uvarum* in SSJ were more attractive to summer morphs. Previous studies indicated equivalent attraction of winter and summer morphs to *S. cerevisiae* but this is contrary to the evidence reported here (Erdei et al. 2022; Kirkpatrick et al. 2018; Wong et al. 2018).

Previous studies revealed that female *D. suzukii* summer morphs exhibit greater attraction towards strawberry juice over apple cider vinegar, with winter morphs showing the opposite response (Clymans et al. 2019), whilst Kirkpatrick et al. (2018) reported female summer morphs were repelled by, and winter-morph females attracted to, geosmin, a compound thought to be indicative of sub-optimal oviposition sites (Stensmyr et al. 2012). Noble et al. (2019), using similar methods to ours, found summer morphs were more attracted to *H. uvarum* than *S. cerevisiae* whereas we found no significant difference. This could be due to several factors, including yeast strain or culture media (Günther et al. 2019; Lasa et al. 2019; Palanca et al. 2013). Additionally, Noble et al. (2019) used ‘live’ yeast rather than metabolite supernatants. Overall, there is a general consensus that both yeast and fruit volatiles are important in attraction of *D. suzukii* (Abraham et al. 2015; Bueno et al. 2020; Jones et al. 2021; Lasa et al. 2019; Scheidler et al. 2015), but there is a lack of understanding of the mechanisms by which formulations of yeasts and fruit products can be optimized to enhance the efficacy of baits. Because yeast attraction can vary between laboratory and field experiments (Jones et al. 2021) and a laboratory strain *D. suzukii* established in 2013 was used for attraction tests, these findings need to be validated with field experiments/ populations. However, these results indicate that *D. suzukii* summer and winter morphs may respond differently to certain environmental cues (see also Clymans et al. 2019; Conroy et al. 2024; Kirkpatrick et al. 2018), and this has the potential to be exploited for novel pest control measures.

Neither of the co-cultured yeasts, *M. pulcherrima* + *H. uvarum* or *H. uvarum* + *C. zemplininia*, were more attractive than their single culture combined counterparts or *H. uvarum* alone. For winter morphs *H. uvarum* + *C. zemplinina* co-culture in SJJ was less attractive than post-culture blends of constituent yeasts, with both co-cultured yeasts being less attractive than *H. uvarum* alone. The prediction that combinations of co-culture yeast would be more attractive than post-culture mixes of single yeasts was therefore not supported. However, here only simple co-cultured combinations of two yeast species were tested. Saccharomycetales yeast communities on common commercial host fruits of *D. suzukii* comprise of 13–35 different yeast types (Jones 2022), and more complex yeast communities could be explored to potentially enhance bait attraction. We did not test how these co-fermented combinations interacted during culture, and it is conceivable that one yeast dominated during the co-culture process. During culture in potato dextrose media broth, *C. zemplininia* took twice as long to reach an optical density of ≥ 1.8 than *H. uvarum* (Scheidler et al. 2015). This could explain the equivalent attraction between *H. uvarum* alone and when co-fermented with *C. zemplininia* but does not explain why *H. uvarum* alone was more attractive to winter morphs than when co-fermented in SSJ with *C. zemplininia*.

Variability in attractiveness across experiments was observed, as *H. uvarum* fermented separately in SSJ was significantly more attractive to summer- than winter-morph flies in one out of three experiments with the reverse result when fermented in YPD. The treatments to which the summer morphs were significantly more attracted than winter morphs should be treated with caution as two of the six water controls had greater activity counts for summer morphs. The data was analysed as proportions to account for the difference in number of flies between winter- and summer-morph experiments. However, this finding may still be a consequence of the reduced activity typical of winter-morph *D. suzukii*. Additionally, YPD media contains a yeast extract and may have influenced the attraction of flies and YPD media on its own proved more attractive that water in five of the six experiments it was used.

Our study used activity counts over 24 h, where flies were allowed to leave and re-visit the baits, as a proxy for attraction (Noble et al. 2019). Other studies testing *D. suzukii* attraction to yeast have used permanent or fatal choice (Bueno et al. 2020; Jones et al. 2021; Lasa et al. 2019; Scheidler et al. 2015) or apparatus which only detects one fly choice per individual e.g. two-way choice tests with T-mazes (Jones et al. 2021). Using counts as a proxy for attraction allows flies the option over the experiment period of making multiple choices if they choose and it may be that flies prefer a range of yeasts when given the option of repeated choices. In addition, our tubes could accommodate more than a single fly, potentially resulting in intra-specific interactions and/or competition. Additionally, by using activity as a proxy for attraction you are assuming that a more active stimulus will cause increased activity, also this method only records counts and not time spent in tubes. It is possible that flies could spend longer in certain tubes due to attraction and this could result in reduced counts. To try and mitigate this the infrared beams that registered the counts were placed close to the baits.

This study identified candidate yeast species and combinations that act as chemo-attractants to female *D. suzukii* winter morphs that also differ in attraction between summer and winter morphs, although preference differing between yeasts was only observed for select yeast and combinations. This potentially has important implications for integrated pest management strategies for this pest and may mean that certain yeast baits could be seasonally tailored to better target the different morphs in attractandkill strategies. Although, from the limited number of yeasts and combinations tested here, ones that differ in attractiveness to the morphs may be in the minority.

## Electronic Supplementary Material

Below is the link to the electronic supplementary material.

Supplementary Material 1

## Data Availability

Retention times, retention indices and relative amounts of components are available in the Supplementary Material. Raw activity count data are available from the corresponding author upon reasonable request.
