# Supplementary material for: Differential Attraction of Summer and Winter Morphs of Spotted Wing Drosophila, Drosophila suzukii, to Yeasts
Source: J Chem Ecol. 2025 Feb 5;51(1):23. doi: 10.1007/s10886-025-01561-x (PMC11799104; doi:10.1007/s10886-025-01561-x)
Supplement: Supplementary file 1 — Supplementary Material 1 [file 10886_2025_1561_MOESM1_ESM.docx]

# Supplementary Material

# Differential Attraction of Summer and Winter Morphs of Spotted Wing Drosophila, *Drosophila suzukii*, to Yeasts

## Rory Jones*^1,2^ Matthew R. Goddard^1^ Paul E. Eady^1^ David R. Hall^3^ Daniel P. Bray^3^ Dudley I. Farman^3^ Michele T. Fountain^2^

^1^School of Life Sciences, University of Lincoln, Lincoln, LN6 7DL, UK.

^2^NIAB, New Road, East Malling, Kent ME19 6BJ, UK.

^3^Natural Resources Institute, University of Greenwich, Chatham Maritime, Kent ME4 4TB, UK.

**Table S1** Origin, source and strain of yeast isolates used in this study.

**Table S2** Choice tests of single yeast species, combinations of singly cultured then combined yeasts and co-cultured combinations cultured separately in sterile strawberry juice (SSJ) or yeast peptone dextrose (YPD) media and tested for attraction to female winter and summer morphs of *Drosophila suzukii*.

**Table S3.** Retention times, retention indices and relative amounts of components, collected by SPME and analyzed by GC-MS on polar DBWax column, produced by the single yeast species grown in yeast sterile strawberry juice (SSJ).

**Table S4.** Retention times, retention indices and relative amounts of components, collected by SPME and analyzed by GC-MS on polar DBWax column, produced by the single yeast species grown in yeast peptone dextrose (YPD).

**Table S5.** Results from Pearson’s correlations between principal components scores (PC1 and PC2) for volatile analyses and activity counts of summer and winter morphs of *Drosophila suzukii* after 4 h and 24 h for yeasts cultured in sterile strawberry juice (SSJ) or yeast peptone dextrose (YPD) media.

**Fig. S1** 32-channel modified LAM10H Locomotor Activity Monitor rig with open-ended tubes.

**Fig. S2** Mean number of activity counts (± SE; *N* = 12), used as a proxy for attraction of female summer morphs of *Drosophila suzukii* to single yeasts.

**Fig. S3** Mean number of activity counts (± SE; *N* = 12), used as a proxy for attraction of female winter morphs of *Drosophila suzukii* to single yeasts.

**Fig. S4** Mean number of activity counts (± SE; *N* = 12) used as a proxy for attraction of summer morphs of *Drosophila suzukii* to combinations of yeasts after post-culture blending of singly cultured yeasts.

**Fig. S5** Mean number of activity counts (± SE; *N* = 12) used as a proxy for attraction of winter morphs of *Drosophila suzukii* to combinations of yeasts after post-culture blending of singly cultured yeasts.

**Fig. S6** Mean number of activity counts (± SE; *N* = 12), used as a proxy for attraction for summer-morph *Drosophila suzukii* tor combinations of co-cultured yeasts.

**Fig. S7** Mean number of activity counts (± SE; *N* = 12), used as a proxy for attraction for winter-morph *Drosophila suzukii* for combinations of co-cultured yeasts.

**Table S1** Origin, source and strain of yeast isolates used in this study.

| Species | Strain | Origin | Source | Reference |
| --- | --- | --- | --- | --- |
| *Hanseniaspora uvarum* | 201 | New Zealand | Chardonnay fruit | Gayevskiy et al. 2012 |
| *Metschnikowia pulcherrima* | 190 | New Zealand | Sauvignon Blanc ferment | Goddard culture collection |
| *Pichia pijperi* | 218 | New Zealand | Pinot noir ferment | Goddard culture collection |
| *Candida zemplininia* | 164 | New Zealand | Chardonnay ferment | Anfang et al. 2009 |
| *Saccharomyces cerevisiae* | EC-1118 | France | Commercial wine yeast | Lallemand Inc. |

**Table S2** Choice tests of single yeast species, combinations of singly cultured then combined yeasts and co-cultured combinations cultured separately in sterile strawberry juice (SSJ) or yeast peptone dextrose (YPD) media and tested for attraction to female winter and summer morphs of *Drosophila suzukii*. Yeasts were compared to water, culture media and a commercial product, Combi-protec (5%).

| Experiment | Single yeasts | | | | Combination singly cultured | | | | | Combination co-cultured | | | |  |
| --- | --- | --- | --- | --- | --- | --- | --- | --- | --- | --- | --- | --- | --- | --- |
| Morphological type | Summer | | Winter | | | Summer | | Winter | | Summer | | Winter | |  |
| Culture media | SSJ | YPD | SSJ | YPD | | SSJ | YPD | SSJ | YPD | SSJ | YPD | SSJ | YPD | |
| Treatment |  |  |  |  | |  |  |  |  |  |  |  |  | |
| *Hanseniaspora uvarum* | ✓ | ✓ | ✓ | ✓ | | ✓ | ✓ | ✓ | ✓ | ✓ | ✓ | ✓ | ✓ | |
| *Metschnikowia pulcherrima* | ✓ | ✓ | ✓ | ✓ | |  |  |  |  |  |  |  |  | |
| *Pichia pijperi* | ✓ | ✓ | ✓ | ✓ | |  |  |  |  |  |  |  |  | |
| *Candida zemplininia* | ✓ | ✓ | ✓ | ✓ | |  |  |  |  |  |  |  |  | |
| *Saccharomyces cerevisiae* | ✓ | ✓ | ✓ | ✓ | |  |  |  |  |  |  |  |  | |
| *H. uvarum + C. zemplininia* |  |  |  |  | | ✓ | ✓ | ✓ | ✓ | ✓ | ✓ | ✓ | ✓ | |
| *M. pulcherrima + H. uvarum* |  |  |  |  | | ✓ | ✓ | ✓ | ✓ | ✓ | ✓ | ✓ | ✓ | |
| *M. pulcherrima + P. pijperi* |  |  |  |  | | ✓ | ✓ | ✓ | ✓ |  |  |  |  | |
| *M. pulcherrima + P. pijperi + H. uvarum* |  |  |  |  | | ✓ | ✓ | ✓ | ✓ |  |  |  |  | |
| *H. uvarum + C. zemplininia* (co-cultured) |  |  |  |  | |  |  |  |  | ✓ | ✓ | ✓ | ✓ | |
| *M. pulcherrima + H. uvarum* (co-cultured) |  |  |  |  | |  |  |  |  | ✓ | ✓ | ✓ | ✓ | |
| Growth media | ✓ | ✓ | ✓ | ✓ | | ✓ | ✓ | ✓ | ✓ | ✓ | ✓ | ✓ | ✓ | |
| Combi-protec | ✓ | ✓ | ✓ | ✓ | | ✓ | ✓ | ✓ | ✓ | ✓ | ✓ | ✓ | ✓ | |
| Distilled water | ✓ | ✓ | ✓ | ✓ | | ✓ | ✓ | ✓ | ✓ | ✓ | ✓ | ✓ | ✓ | |

**Table S3.** Retention times, retention indices and relative amounts (% TIC peak area) of components, collected by SPME and analyzed by GC-MS on polar DBWax column, produced by the single yeast species *Hanseniaspora uvarum* (Hu), *Metschnikowia pulcherrima* (Mp), *Pichia pijperi* (Pp), *Candida zemplininia* (Cz) and *Saccharomyces cerevisiae* (Sc) grown in sterile strawberry juice (SSJ). TIC peak areas were used in the PCA but relative percentages are shown here for clarity along with total counts relative to those for SSJ (Retention indices relative to retention times of n-alkanes; compounds identified by comparison of mass spectra and retention times with those of authentic standards except those designated “?” which were identified by mass spectra only).

|  |  |  | SSJ (*N*=3) | | Hu (*N*=4) | | Mp (*N*=4) | | Pp (*N*=4) | | Cz (*N*=3) | | Sc (*N*=3) | |
| --- | --- | --- | --- | --- | --- | --- | --- | --- | --- | --- | --- | --- | --- | --- |
| RT(min) | RI | Compound | mean | SE | mean | SE | mean | SE | mean | SE | mean | SE | mean | SE |
| 2.22 |  | ethyl acetate | 0.0 | 0.0 | 42.0 | 1.7 | 20.3 | 0.8 | 15.1 | 0.7 | 0.0 | 0.0 | 0.9 | 0.9 |
| 2.65 |  | ethanol | 27.3 | 4.0 | 17.2 | 1.1 | 28.3 | 3.0 | 28.9 | 1.2 | 46.7 | 5.2 | 37.0 | 3.3 |
| 3.78 | 1039 | unknown? | 0.0 | 0.0 | 18.7 | 1.8 | 0.0 | 0.0 | 0.0 | 0.0 | 17.0 | 3.3 | 0.0 | 0.0 |
| 4.42 | 1087 | hydrocarbon? | 0.0 | 0.0 | 0.2 | 0.1 | 3.2 | 0.1 | 1.5 | 0.6 | 0.8 | 0.2 | 2.1 | 0.1 |
| 4.78 | 1114 | 2/3-methylbutyl acetate | 0.0 | 0.0 | 1.1 | 0.4 | 0.0 | 0.0 | 7.3 | 2.7 | 0.0 | 0.0 | 2.6 | 1.0 |
| 5.39 | 1160 | alcohol? | 0.0 | 0.0 | 0.0 | 0.0 | 0.0 | 0.0 | 0.0 | 0.0 | 3.1 | 0.6 | 0.7 | 0.7 |
| 5.68 | 1182 | methyl hexanoate | 11.2 | 1.3 | 0.0 | 0.0 | 0.0 | 0.0 | 0.0 | 0.0 | 0.0 | 0.0 | 0.0 | 0.0 |
| 6.03 | 1208 | 2/3-methylbutanol | 0.4 | 0.4 | 8.5 | 0.9 | 18.3 | 0.8 | 15.5 | 1.3 | 5.4 | 0.7 | 16.6 | 1.6 |
| 6.35 | 1232 | ethyl hexanoate | 9.8 | 1.4 | 0.3 | 0.2 | 0.0 | 0.0 | 5.7 | 0.4 | 1.3 | 0.1 | 7.0 | 0.4 |
| 6.60 | 1251 | styrene | 0.0 | 0.0 | 0.0 | 0.0 | 0.0 | 0.0 | 4.9 | 0.2 | 0.0 | 0.0 | 9.7 | 1.1 |
| 6.88 | 1272 | hexyl acetate | 0.3 | 0.3 | 0.0 | 0.0 | 0.0 | 0.0 | 0.5 | 0.3 | 2.5 | 0.4 | 0.0 | 0.0 |
| 6.99 | 1280 | alcohol? | 0.7 | 0.7 | 0.8 | 0.1 | 0.2 | 0.2 | 0.4 | 0.2 | 7.2 | 0.4 | 0.5 | 0.2 |
| 7.07 | 1286 | acetoin | 0.0 | 0.0 | 0.0 | 0.0 | 0.0 | 0.0 | 0.0 | 0.0 | 0.0 | 0.0 | 0.0 | 0.0 |
| 7.75 | 1337 | (*E*)-2-hexenyl acetate | 1.1 | 0.6 | 0.0 | 0.0 | 0.0 | 0.0 | 0.0 | 0.0 | 0.0 | 0.0 | 0.0 | 0.0 |
| 7.92 | 1349 | ethyl 2-hexenoate | 0.0 | 0.0 | 0.0 | 0.0 | 0.0 | 0.0 | 0.0 | 0.0 | 0.0 | 0.0 | 0.0 | 0.0 |
| 8.05 | 1359 | hexanol | 0.0 | 0.0 | 1.3 | 0.1 | 5.0 | 0.2 | 1.3 | 0.1 | 1.9 | 0.1 | 1.9 | 0.1 |
| 8.75 | 1412 | (*E*)-2-hexenol | 0.6 | 0.6 | 0.0 | 0.0 | 0.0 | 0.0 | 0.0 | 0.0 | 0.0 | 0.0 | 0.0 | 0.0 |
| 8.97 | 1430 | di-tertbutyl-benzene | 0.0 | 0.0 | 0.0 | 0.0 | 0.0 | 0.0 | 0.0 | 0.0 | 0.0 | 0.0 | 0.0 | 0.0 |
| 9.10 | 1440 | ethyl octanoate | 0.0 | 0.0 | 0.0 | 0.0 | 0.0 | 0.0 | 1.0 | 0.2 | 0.0 | 0.0 | 1.3 | 0.1 |
| 9.25 | 1452 | acetic acid | 0.0 | 0.0 | 1.3 | 0.1 | 0.0 | 0.0 | 0.0 | 0.0 | 0.4 | 0.4 | 0.8 | 0.4 |
| 10.21 | 1529 | benzaldehyde | 14.3 | 0.8 | 1.6 | 0.1 | 4.3 | 0.7 | 0.0 | 0.0 | 1.3 | 0.7 | 1.1 | 0.6 |
| 10.53 | 1554 | linalool | 17.7 | 1.7 | 3.0 | 0.3 | 8.8 | 1.3 | 3.5 | 0.5 | 5.0 | 0.2 | 4.8 | 0.7 |
| 11.14 | 1604 | strawberry furan | 7.1 | 0.3 | 1.2 | 0.1 | 3.6 | 0.5 | 1.4 | 0.2 | 2.0 | 0.2 | 1.9 | 0.2 |
| 11.60 | 1644 | ethyl decanoate | 0.0 | 0.0 | 0.0 | 0.0 | 0.0 | 0.0 | 0.0 | 0.0 | 0.0 | 0.0 | 0.0 | 0.0 |
| 11.75 | 1658 | phenylacetaldehyde | 4.6 | 0.1 | 0.8 | 0.3 | 1.9 | 0.7 | 0.6 | 0.4 | 1.2 | 0.6 | 2.0 | 0.4 |
| 11.91 | 1672 | 2/3-methyl butanoic acid | 1.3 | 1.3 | 0.6 | 0.0 | 2.0 | 0.2 | 0.7 | 0.1 | 1.2 | 0.7 | 1.1 | 0.1 |
| 12.34 | 1710 | terpineol | 0.0 | 0.0 | 0.0 | 0.0 | 0.0 | 0.0 | 0.0 | 0.0 | 0.0 | 0.0 | 0.0 | 0.0 |
| 12.65 | 1738 | benzyl acetate | 0.4 | 0.4 | 0.0 | 0.0 | 0.0 | 0.0 | 0.0 | 0.0 | 0.0 | 0.0 | 0.0 | 0.0 |
| 13.62 | 1826 | 2-phenylethyl acetate | 0.0 | 0.0 | 0.0 | 0.0 | 0.0 | 0.0 | 1.6 | 0.5 | 0.0 | 0.0 | 0.5 | 0.2 |
| 13.86 | 1849 | hexanoic acid | 3.2 | 0.3 | 0.4 | 0.1 | 1.2 | 0.2 | 0.8 | 0.1 | 0.9 | 0.1 | 0.9 | 0.1 |
| 14.63 | 1923 | 2-phenylethanol | 0.0 | 0.0 | 1.1 | 0.1 | 2.7 | 0.4 | 9.0 | 1.3 | 2.1 | 0.3 | 6.3 | 1.0 |
| 15.88 | 2048 | nerolidol | 0.0 | 0.0 | 0.0 | 0.0 | 0.0 | 0.0 | 0.0 | 0.0 | 0.0 | 0.0 | 0.0 | 0.0 |
| 16.01 | 2061 | octanoic acid | 0.0 | 0.0 | 0.0 | 0.0 | 0.0 | 0.0 | 0.2 | 0.2 | 0.0 | 0.0 | 0.2 | 0.2 |
| 16.78 | 2130 | ethyl cinnamate | 0.0 | 0.0 | 0.0 | 0.0 | 0.0 | 0.0 | 0.0 | 0.0 | 0.0 | 0.0 | 0.0 | 0.0 |
|  |  |  |  |  |  |  |  |  |  |  |  |  |  |  |
| Total counts relative to SSJ | | | 1.0 |  | 5.0 |  | 1.7 |  | 5.0 |  | 2.9 |  | 3.6 |  |

**Table S4.** Retention times, retention indices and relative amounts (% TIC peak area) of components, collected by SPME and analyzed by GC-MS on polar DBWax column, produced by the single yeast species *Hanseniaspora uvarum* (Hu), *Metschnikowia pulcherrima* (Mp), *Pichia pijperi* (Pp), *Candida zemplininia* (Cz) and *Saccharomyces cerevisiae* (Sc) grown in yeast peptone dextrose (YPD). TIC peak areas were used in the PCA but relative percentages are shown here for clarity along with total counts relative to those for YPD (Retention indices relative to retention times of n-alkanes; compounds identified by comparison of mass spectra and retention times with those of authentic standards except those designated “?” which were identified by mass spectra only).

|  |  |  | YPD (*N* = 3) | | Hu (*N* = 3) | | Mp (*N* = 3) | | Pp (*N* = 3) | | Cz (*N* = 3) | | Sc (*N* = 3) | |
| --- | --- | --- | --- | --- | --- | --- | --- | --- | --- | --- | --- | --- | --- | --- |
| RT(min) | RI | Compound | mean | SE | mean | SE | mean | SE | mean | SE | mean | SE | mean | SE |
| 2.15 | 933 | ethyl acetate | 0.0 | 0.0 | 7.5 | 1.7 | 6.9 | 0.7 | 37.8 | 2.4 | 3.3 | 1.0 | 0.8 | 0.4 |
| 2.55 | 964 | ethanol | 0.0 | 0.0 | 15.5 | 1.8 | 16.7 | 1.7 | 11.5 | 1.3 | 19.2 | 1.3 | 21.3 | 0.1 |
| 3.43 | 1032 | ethyl butanoate | 0.0 | 0.0 | 0.0 | 0.0 | 0.2 | 0.2 | 4.0 | 0.8 | 0.0 | 0.0 | 0.0 | 0.0 |
| 4.14 | 1087 | 3-methyl-2-butenal | 0.0 | 0.0 | 0.1 | 0.1 | 0.0 | 0.0 | 0.0 | 0.0 | 0.0 | 0.0 | 0.0 | 0.0 |
| 4.28 | 1098 | undecane | 0.0 | 0.0 | 1.8 | 0.3 | 2.6 | 0.1 | 0.7 | 0.3 | 3.9 | 0.3 | 0.5 | 0.3 |
| 4.44 | 1110 | 2/3-methylbutyl acetate | 0.0 | 0.0 | 0.8 | 0.1 | 0.5 | 0.3 | 2.2 | 0.4 | 0.3 | 0.2 | 0.5 | 0.2 |
| 4.67 | 1128 | t-butyloxyethanol? | 0.0 | 0.0 | 0.4 | 0.2 | 0.1 | 0.1 | 0.1 | 0.1 | 0.0 | 0.0 | 0.3 | 0.1 |
| 4.84 | 1141 | 2-methyl-2-pentenal? | 0.0 | 0.0 | 0.2 | 0.2 | 0.0 | 0.0 | 0.0 | 0.0 | 0.0 | 0.0 | 0.1 | 0.0 |
| 4.91 | 1147 | butanol | 14.6 | 1.8 | 1.0 | 0.1 | 1.2 | 0.1 | 0.3 | 0.2 | 0.2 | 0.0 | 0.4 | 0.0 |
| 5.02 | 1155 | hydrocarbon | 0.0 | 0.0 | 0.0 | 0.0 | 0.0 | 0.0 | 0.5 | 0.3 | 0.0 | 0.0 | 0.0 | 0.0 |
| 5.20 | 1169 | 4,4-dimethyl-2-pentenal? | 0.0 | 0.0 | 1.4 | 0.7 | 0.5 | 0.2 | 0.9 | 0.0 | 0.3 | 0.3 | 1.7 | 0.4 |
| 5.67 | 1205 | 4-methyl-2-heptanone? | 0.0 | 0.0 | 0.2 | 0.2 | 0.0 | 0.0 | 0.1 | 0.1 | 0.0 | 0.0 | 0.3 | 0.1 |
| 5.85 | 1219 | 2/3-methylbutanol | 0.0 | 0.0 | 59.1 | 1.5 | 63.8 | 0.2 | 38.0 | 1.7 | 56.6 | 1.6 | 64.5 | 1.0 |
| 6.69 | 1282 | 2,6-dimethyl-3-heptanone? | 14.4 | 1.5 | 0.1 | 0.1 | 0.4 | 0.0 | 0.3 | 0.0 | 0.4 | 0.0 | 0.2 | 0.1 |
| 6.82 | 1292 | acetoin | 0.0 | 0.0 | 0.4 | 0.2 | 1.3 | 0.2 | 0.1 | 0.0 | 1.2 | 0.0 | 0.5 | 0.1 |
| 7.41 | 1336 | dimethylpyrazine | 8.1 | 0.2 | 0.1 | 0.1 | 0.3 | 0.0 | 0.1 | 0.0 | 0.0 | 0.0 | 0.2 | 0.1 |
| 7.59 | 1350 | ethyl (E)-2-hexenoate | 0.0 | 0.0 | 0.0 | 0.0 | 0.0 | 0.0 | 0.2 | 0.1 | 0.0 | 0.0 | 0.0 | 0.0 |
| 7.61 | 1352 | ethyl lactate | 0.0 | 0.0 | 0.0 | 0.0 | 0.0 | 0.0 | 0.0 | 0.0 | 0.0 | 0.0 | 0.0 | 0.0 |
| 7.80 | 1366 | hexanol | 0.0 | 0.0 | 0.0 | 0.0 | 0.0 | 0.0 | 0.0 | 0.0 | 0.0 | 0.0 | 0.0 | 0.0 |
| 8.95 | 1457 | acetic acid | 13.7 | 6.3 | 3.1 | 0.3 | 0.2 | 0.2 | 0.3 | 0.1 | 7.9 | 0.9 | 0.5 | 0.2 |
| 9.03 | 1463 | ethyldimethylpyrazine/methyl hydroxy-benzaldehyde | 8.2 | 0.1 | 1.7 | 0.2 | 2.7 | 0.2 | 1.2 | 0.1 | 0.7 | 0.1 | 2.6 | 0.1 |
| 9.89 | 1533 | benzaldehyde | 7.1 | 2.1 | 0.1 | 0.1 | 0.1 | 0.1 | 0.0 | 0.0 | 0.6 | 0.0 | 0.3 | 0.1 |
| 10.40 | 1575 | isobutyric acid | 2.0 | 2.1 | 0.5 | 0.0 | 0.0 | 0.0 | 0.0 | 0.0 | 0.8 | 0.1 | 0.3 | 0.1 |
| 11.11 | 1636 | butanoic acid | 1.2 | 1.3 | 0.5 | 0.0 | 0.0 | 0.0 | 0.0 | 0.0 | 0.8 | 0.1 | 0.3 | 0.1 |
| 11.42 | 1663 | phenylacetaldehyde | 9.5 | 1.1 | 0.7 | 0.2 | 0.5 | 0.1 | 0.2 | 0.0 | 0.7 | 0.0 | 0.9 | 0.1 |
| 11.60 | 1679 | 3-methylbutyric acid | 12.6 | 0.3 | 0.4 | 0.0 | 0.0 | 0.0 | 0.2 | 0.0 | 0.6 | 0.1 | 0.5 | 0.2 |
| 12.22 | 1735 | methionol | 0.0 | 0.0 | 0.1 | 0.1 | 0.0 | 0.0 | 0.0 | 0.0 | 0.1 | 0.1 | 0.0 | 0.0 |
| 13.25 | 1829 | 2-phenylethyl acetate | 0.0 | 0.0 | 0.0 | 0.0 | 0.0 | 0.0 | 0.1 | 0.1 | 0.0 | 0.0 | 0.0 | 0.0 |
| 14.29 | 1931 | 2-phenylethanol | 0.0 | 0.0 | 3.4 | 0.6 | 1.6 | 0.2 | 0.9 | 0.5 | 2.2 | 0.3 | 3.1 | 0.1 |
| 17.92 | 2323 | BHT | 8.7 | 1.8 | 0.8 | 0.1 | 0.4 | 0.1 | 0.2 | 0.2 | 0.5 | 0.3 | 0.4 | 0.0 |
|  |  |  |  |  |  |  |  |  |  |  |  |  |  |  |
| Total counts relative to YPD | | | 1.0 |  | 28.4 |  | 29.7 |  | 51.8 |  | 30.7 |  | 31.0 |  |

**Table S5** Results from Pearson’s correlations between principal components scores (PC1 and PC2) for volatile analyses and activity counts of summer and winter morphs of *Drosophila suzukii* after 4 h and 24 h for yeasts cultured in sterile strawberry juice (SSJ) or yeast peptone dextrose (YPD) media.

|  |  |  | 4 hours | | | 24 hours | | |
| --- | --- | --- | --- | --- | --- | --- | --- | --- |
| PC | Culture media | Morph | *r* | *df* | *P* | *r* | *df* | *P* |
| PC1 | | | | | | | | |
|  | SSJ | summer | 0.05 | 16.00 | 0.83 | 0.30 | 16.00 | 0.23 |
|  |  | winter | -0.33 | 16.00 | 0.18 | -0.29 | 16.00 | 0.25 |
|  | YPD | summer | 0.43 | 16.00 | 0.08 | 0.14 | 16.00 | 0.57 |
|  |  | winter | 0.16 | 16.00 | 0.53 | 0.22 | 16.00 | 0.39 |
| PC2 | | | | | | | | |
|  | SSJ | summer | -0.14 | 16.00 | 0.57 | -0.22 | 16.00 | 0.39 |
|  |  | winter | -0.27 | 16.00 | 0.28 | -0.23 | 16.00 | 0.35 |
|  | YPD | summer | -0.03 | 16.00 | 0.91 | -0.36 | 16.00 | 0.14 |
|  |  | winter | -0.23 | 16.00 | 0.36 | -0.35 | 16.00 | 0.15 |


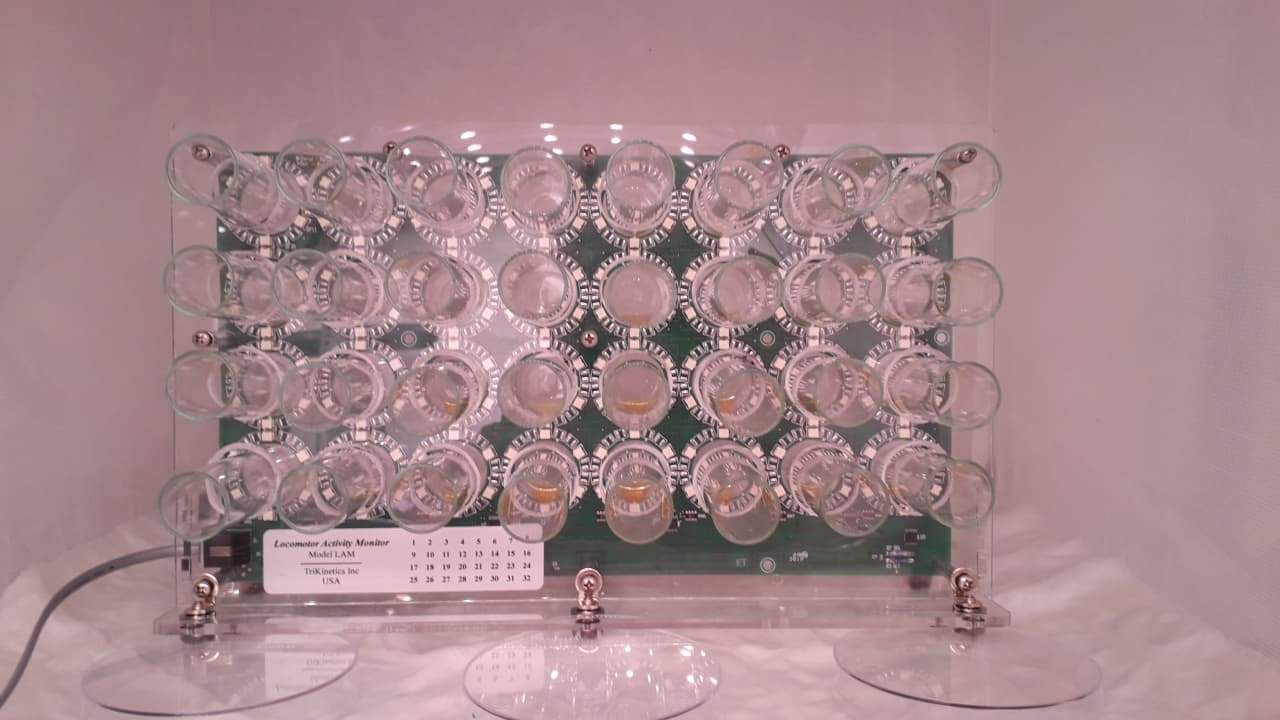


**Fig. S1** 32-channel modified LAM10H Locomotor Activity Monitor rig with open-ended tubes. Modification involved enlarging the holes in the front sheet of Perspex so that they could accommodate 25mm diameter tubes. The activity monitor was set up at angle of 20° from horizontal to ensure that the baits in the tubes did not pass the 23 mm mark on the tube which was where the infra-red beams were positioned. Tubes contained 0.2 mL of supernatant containing yeast metabolites, YPD or sterile strawberry juice media, Combi-protec or water controls. Each of eight treatments was present in each row in a random order to give four replicates per run.


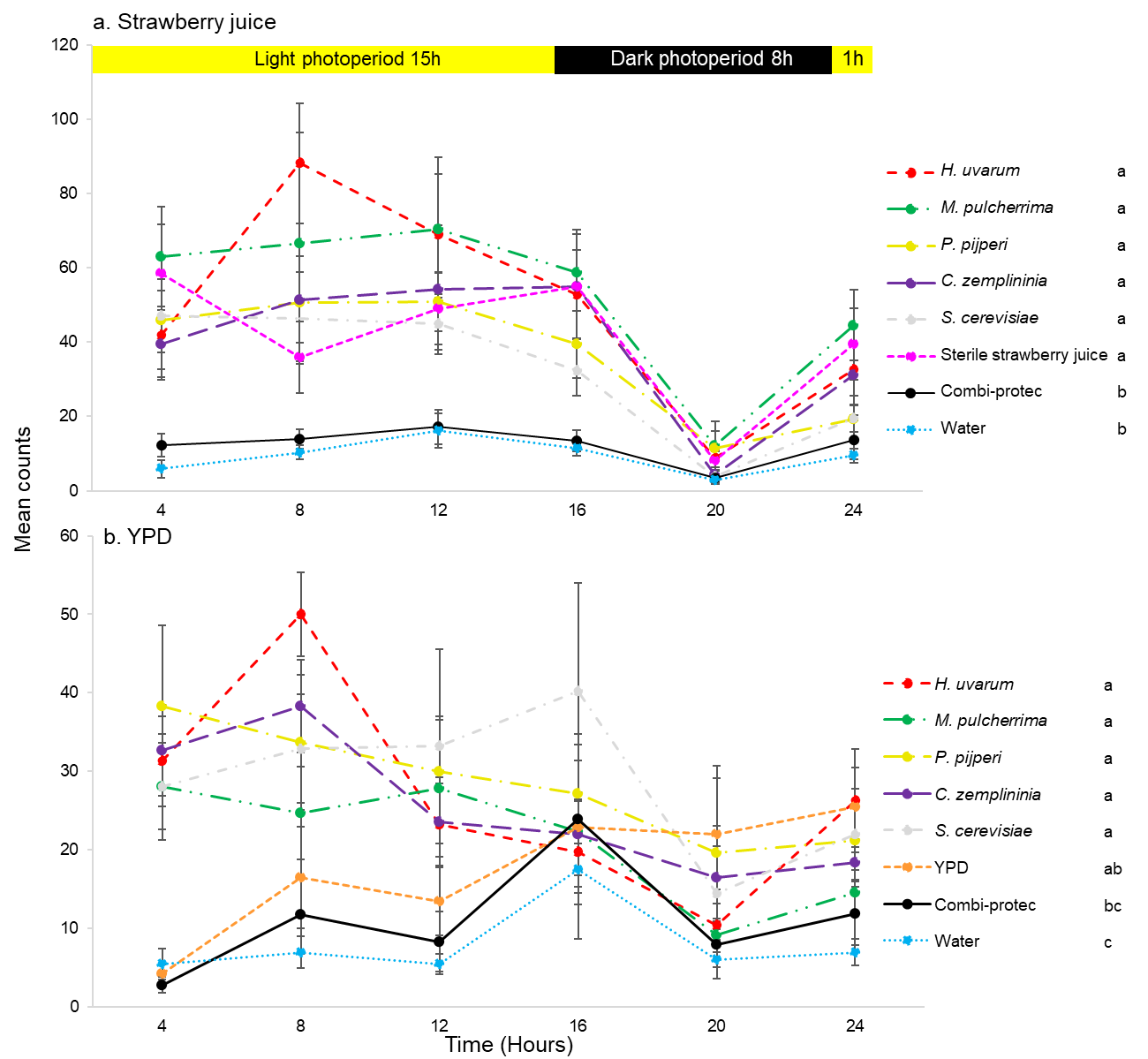


**Fig. S2** Mean number of activity counts (± SE; *N* = 12), used as a proxy for attraction of female summer morphs of *Drosophila suzukii* to single yeasts, cultured in (a) sterile strawberry juice and (b) YPD media, culture media controls (sterile strawberry juice or YPD), Combi-protec and distilled water controls. The colored bar at the top of the graph represents the timing of the 16: 8 Light (yellow bar): dark (black bar) photoperiod over the duration of the experiments. Different letters next to treatment names show significance differences in attraction between treatments, determined by Tukey.


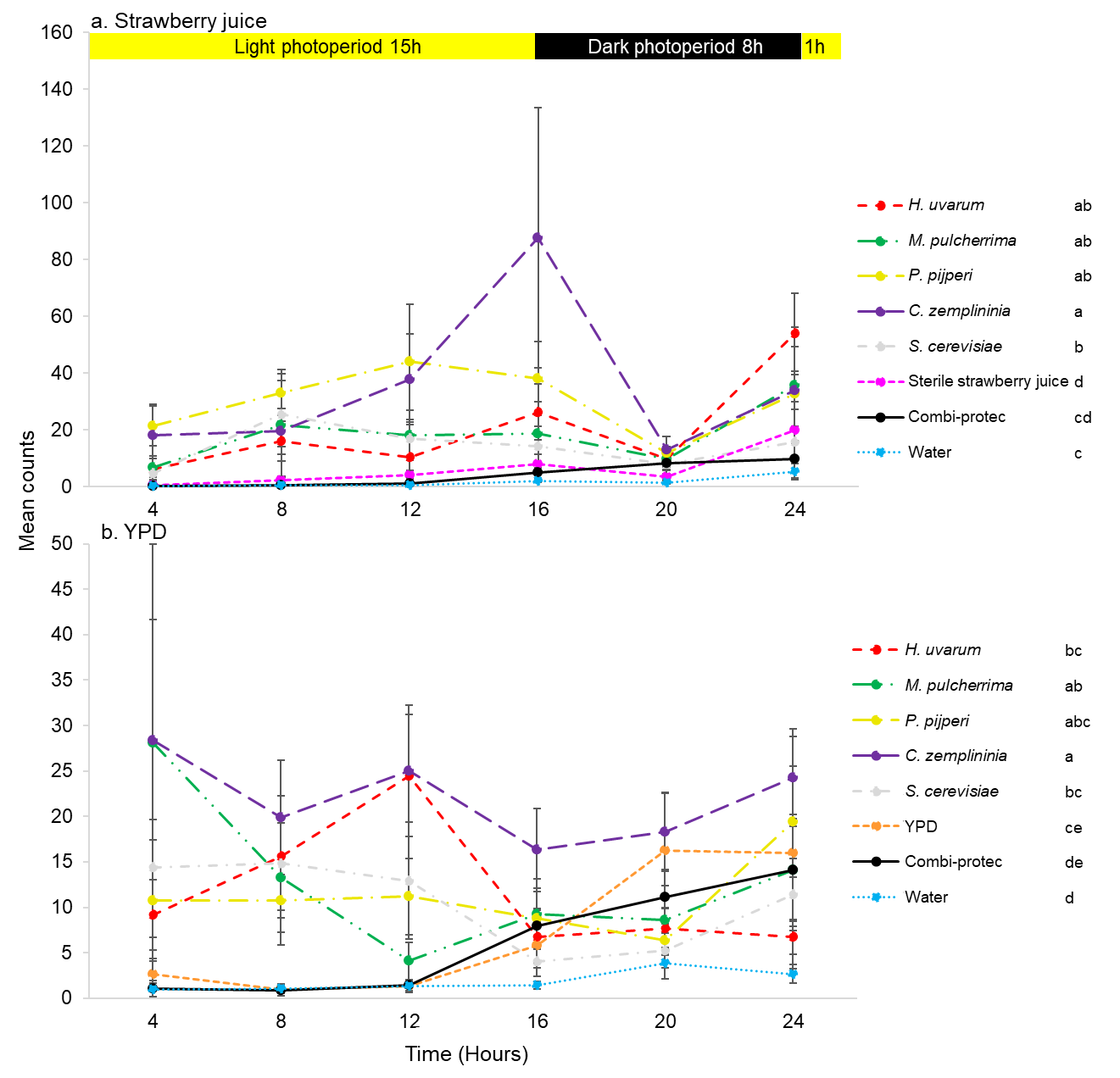


**Fig. S3** Mean number of activity counts (± SE; *N* = 12), used as a proxy for attraction of female winter morphs of *Drosophila suzukii* to single yeasts, cultured in (a) sterile strawberry juice and (b) YPD media, culture media controls (sterile strawberry juice or YPD), Combi-protec and distilled water controls. The colored bar at the top of the graph represents the timing of the 16: 8 Light (yellow bar): dark (black bar) photoperiod over the duration of the experiments. Different letters next to treatment names show significance differences in attraction between treatments, determined by Tukey.


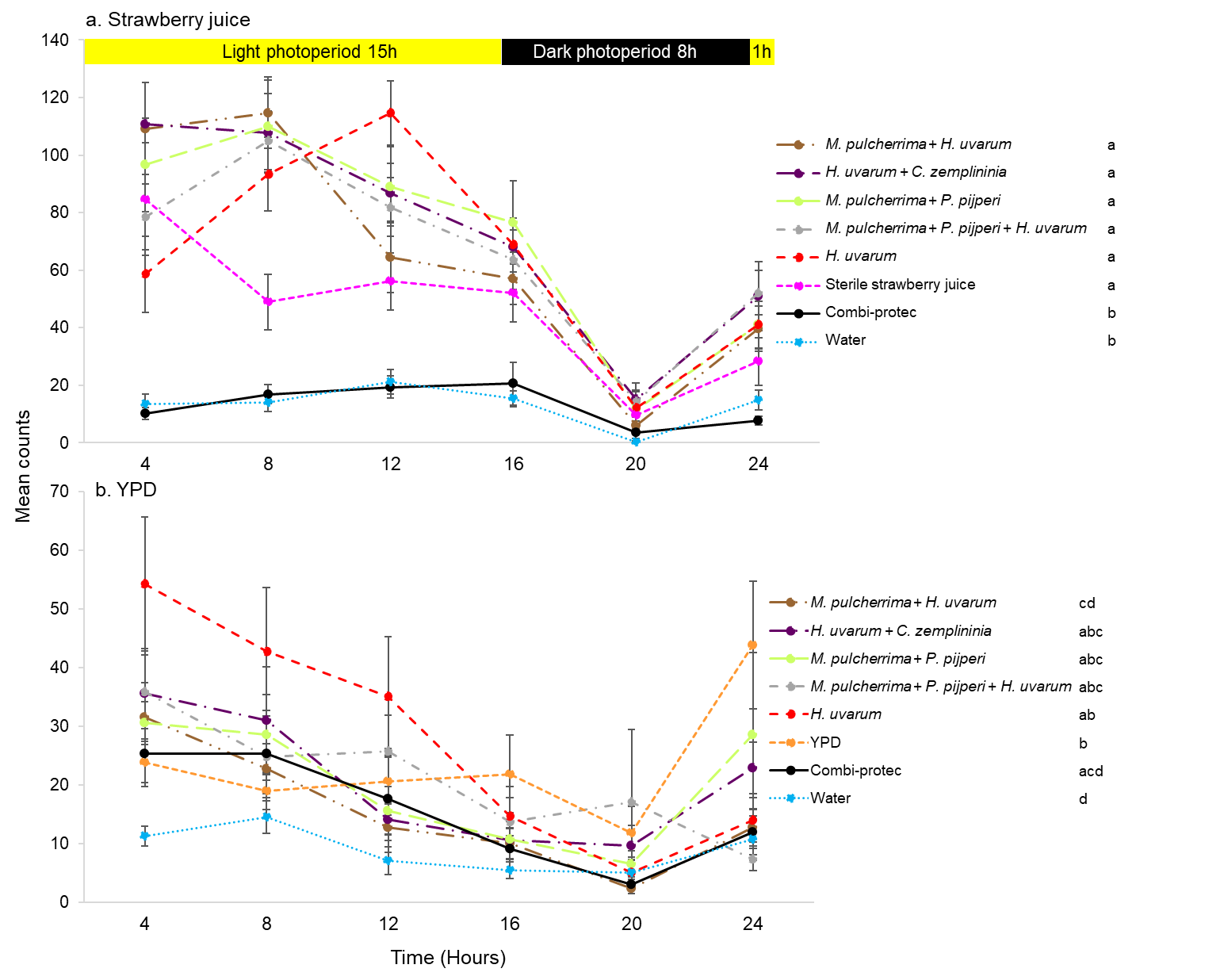


**Fig. S4** Mean number of activity counts (± SE; *N* = 12) used as a proxy for attraction of summer morphs of *Drosophila suzukii* to combinations of yeasts after post-culture blending of singly cultured yeasts cultured in (a) sterile strawberry juice and (b) yeast peptone dextrose (YPD) media, single yeast *H. uvarum*, culture media controls (sterile strawberry juice or YPD), Combi-protec and distilled water controls. The colored bar at the top of the graph represents the timing of the 16: 8 Light (yellow bar): dark (black bar) photoperiod over the duration of the experiments. Different letters next to treatment names show significance differences in attraction between treatments, determined by Tukey.

**
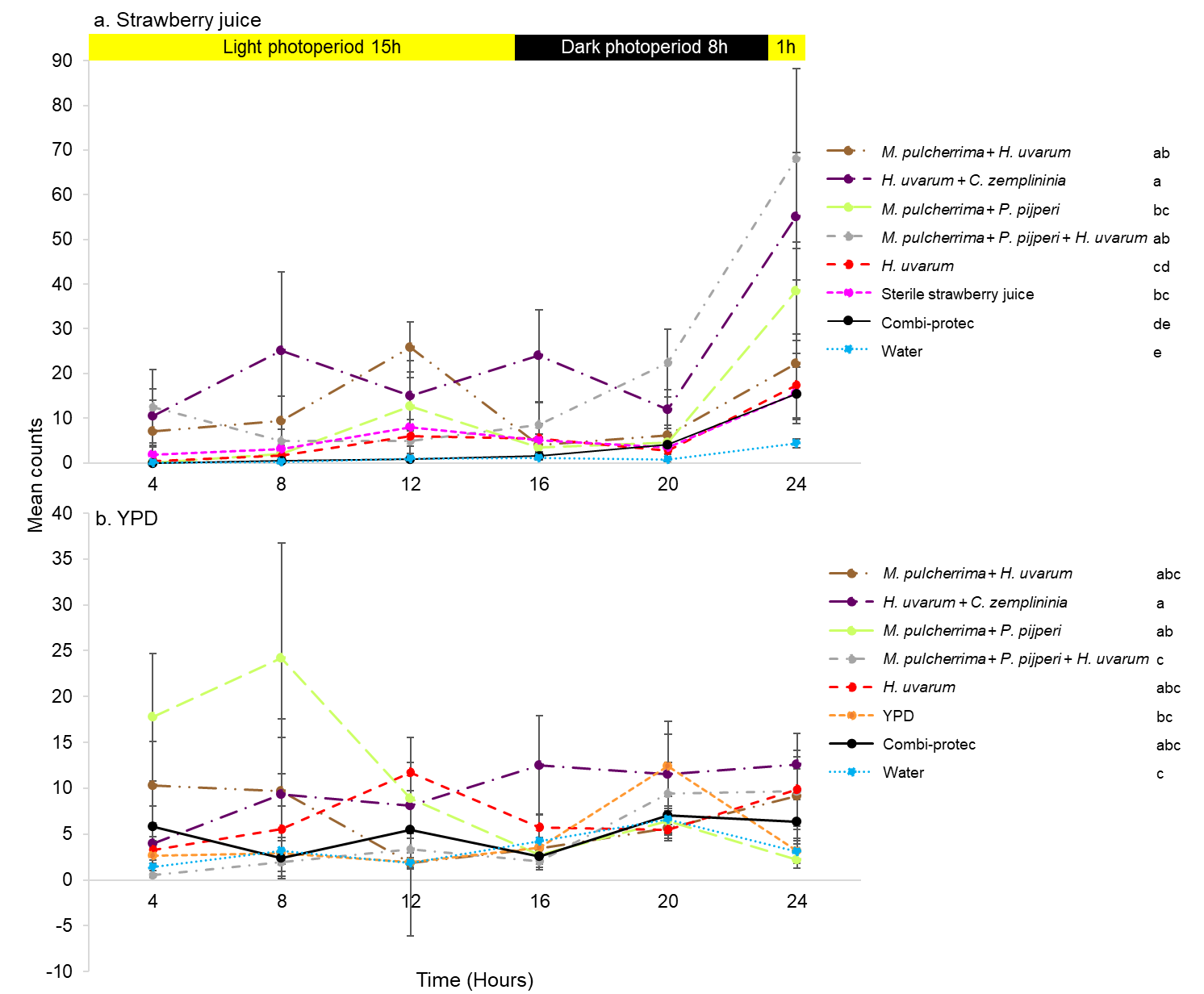
**

**Fig. S5** Mean number of activity counts (± SE; *N* = 12) used as a proxy for attraction of winter morphs of *Drosophila suzukii* to combinations of yeasts after post-culture blending of singly cultured yeasts cultured in (a) sterile strawberry juice and (b) yeast peptone dextrose (YPD) media, single yeast *H. uvarum*, culture media controls (sterile strawberry juice or YPD), Combi-protec and distilled water controls. The colored bar at the top of the graph represents the timing of the 16: 8 Light (yellow bar): dark (black bar) photoperiod over the duration of the experiments. Different letters next to treatment names show significance differences in attraction between treatments, determined by Tukey.


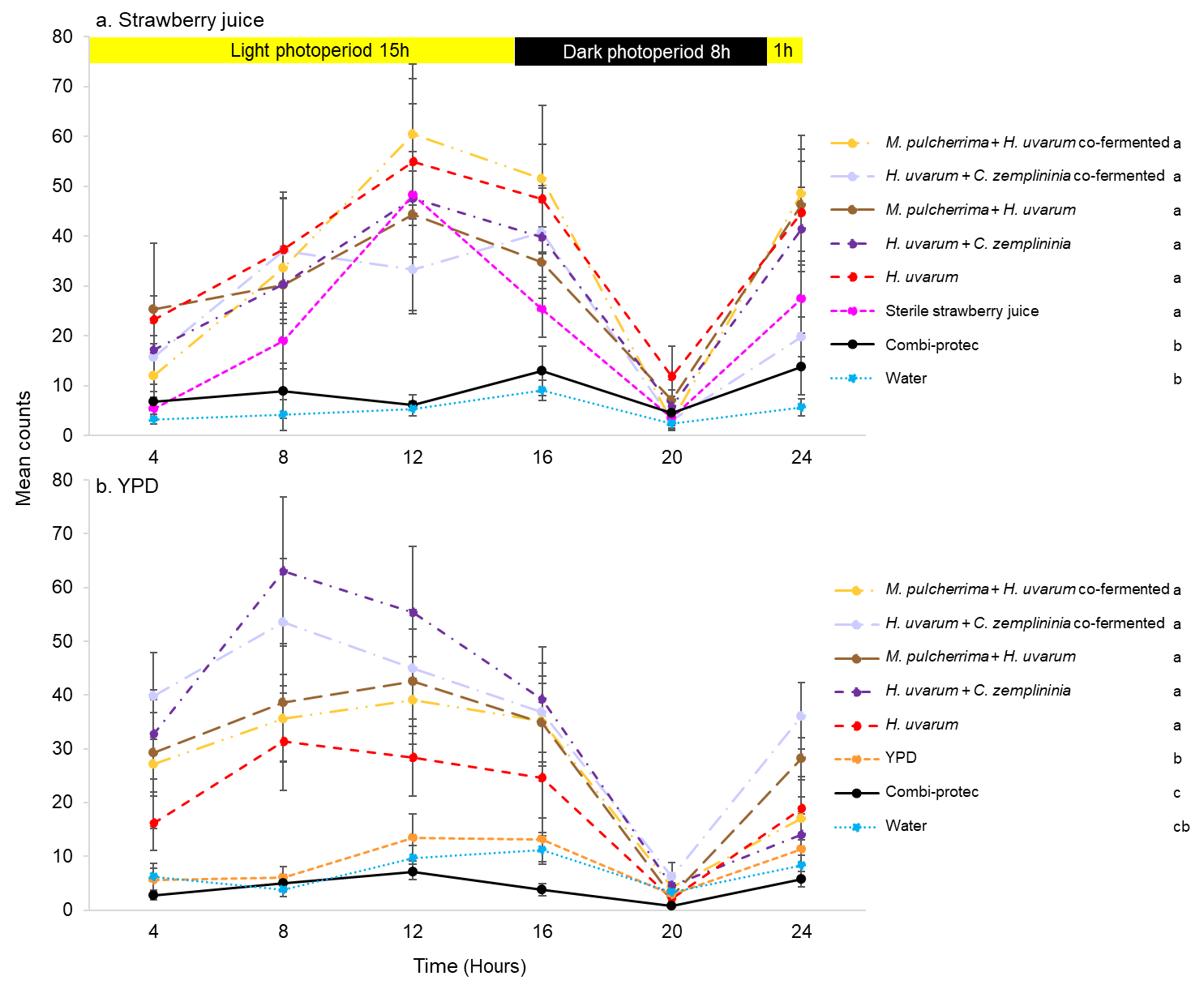


**Fig. S6** Mean number of activity counts (± SE; *N* = 12), used as a proxy for attraction for summer-morph *Drosophila suzukii* for combinations of co-cultured yeasts (*M. pulcherrima + H. uvarum* and *H. uvarum + C. zemplininia*) grown in (a). sterile strawberry juice and (b) yeast peptone dextrose (YPD) media, alongside their singly cultured and combined counterparts as well as single yeast *H. uvarum,* growth media control (either sterile strawberry juice of YPD), Combi-protec and negative control distilled water. Co-cultured combinations were created by inoculating cultures with equal numbers of cells (totalling 1x10^6^ cells per mL) from each yeast species. The colored bar at the top of the graph represents the timing of the 16: 8 Light (yellow bar): dark (black bar) photoperiod over the duration of the experiments. Different letters next to treatment names show significance differences in attraction between treatments, determined by Tukey.


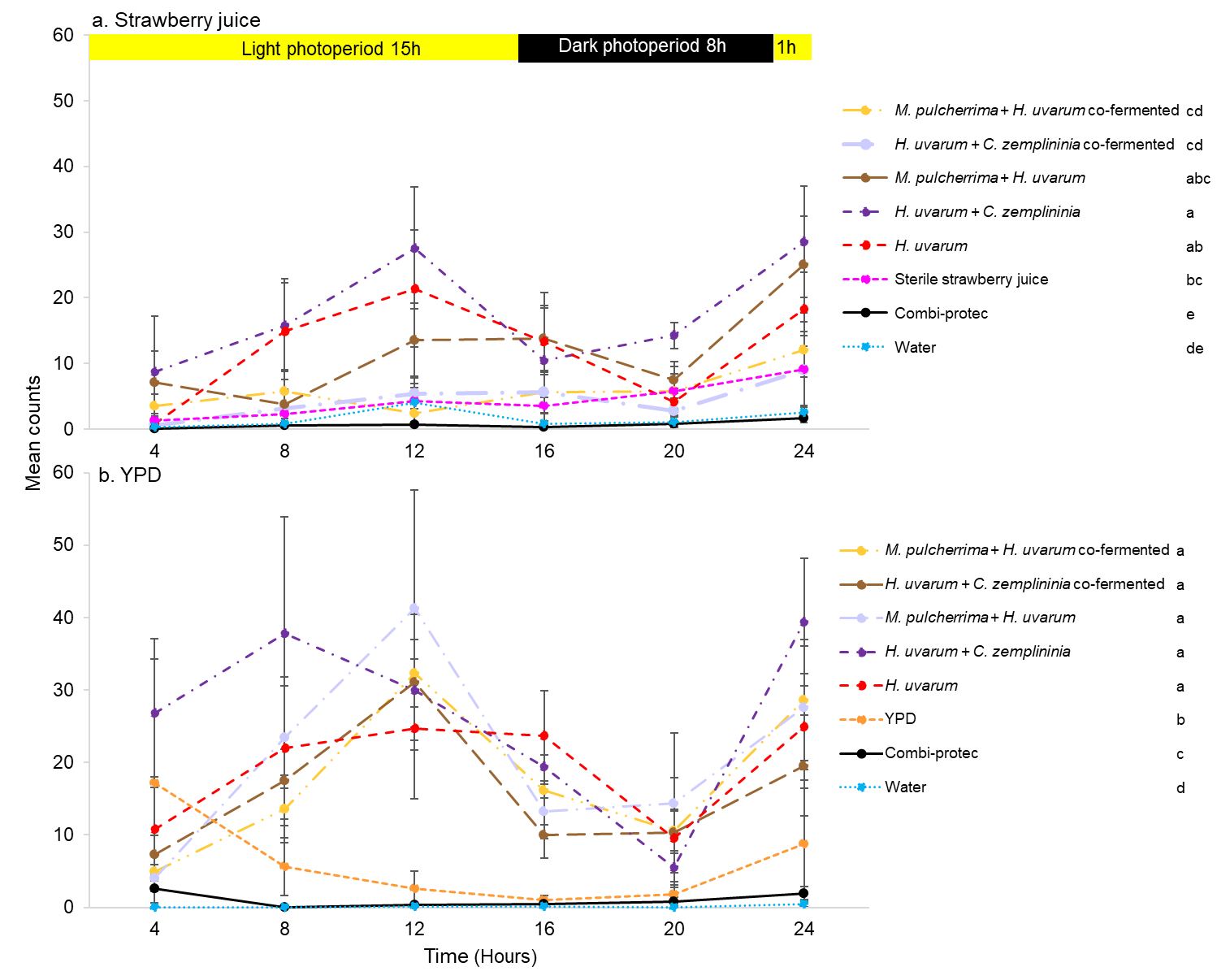


**Fig. S7** Mean number of activity counts (± SE; *N* = 12), used as a proxy for attraction for winter-morph *Drosophila suzukii* for combinations of co-cultured yeasts (*M. pulcherrima + H. uvarum* and *H. uvarum + C. zemplininia*) grown in (a). sterile strawberry juice and (b) yeast peptone dextrose (YPD) media, alongside their singly cultured and combined counterparts as well as single yeast *H. uvarum,* growth media control (either sterile strawberry juice of YPD), Combi-protec and negative control distilled water. The data from the separate experiment was again analyzed, this time separately (i.e. winter and summer morphs not combined) to determine the effect of co-culturing on attraction. Co-cultured combinations were created by inoculating cultures with equal numbers of cells (totalling 1x10^6^ cells per mL) from each yeast species. The colored bar at the top of the graph represents the timing of the 16: 8 Light (yellow bar): dark (black bar) photoperiod over the duration of the experiments. Different letters next to treatment names show significance differences in attraction between treatments, determined by Tukey.
